# Design, Kinematic Optimization, and Evaluation of a Teleoperated System for Middle Ear Microsurgery

**DOI:** 10.1100/2012/907372

**Published:** 2012-08-13

**Authors:** Mathieu Miroir, Yann Nguyen, Jérôme Szewczyk, Olivier Sterkers, Alexis Bozorg Grayeli

**Affiliations:** ^1^Sorbonne Paris Cité, INSERM UMR-S 867, Université Paris Diderot, 75018 Paris, France; ^2^ISIR, CNRS UMR 7222, Université Pierre et Marie Curie, 75005 Paris, France; ^3^Service d'Oto-Rhino-Laryngologie et de Chirurgie Cervico-Faciale, Hôpital Beaujon, AP-HP, 92110 Clichy, France

## Abstract

Middle ear surgery involves the smallest and the most fragile bones of the human body. Since microsurgical gestures and a submillimetric precision are required in these procedures, the outcome can be potentially improved by robotic assistance. Today, there is no commercially available device in this field. Here, we describe a method to design a teleoperated assistance robotic system dedicated to the middle ear surgery. Determination of design specifications, the kinematic structure, and its optimization are detailed. The robot-surgeon interface and the command modes are provided. Finally, the system is evaluated by realistic tasks in experimental dedicated settings and in human temporal bone specimens.

## 1. Introduction

Surgical robotics was born in the mid-eighties and has now extended to nearly all surgical specialties. Its main goal is to enhance surgeon's motor capabilities in terms of movement and force accuracy or to help perform motor tasks which are impossible with the human hand. Several robots have achieved these objectives. The first report of a surgical procedure was in the neurosurgical field. The industrial robot PUMA 560 was used to perform a stereotactic brain biopsy [1]. During the same period, a robot dedicated to this task was developed and commercialized: NeuroMate (Schaerer, Mayfield) [2, 3]. Orthopedic surgery can also benefit from robotic assistance [4, 5]. Projects in this field led to the commercialization of Robodoc (Curexo Technology Corporation, Fremont, CA, USA). More recently, robotic technology was applied to minimally invasive surgery with 2 teleoperated systems: Zeus (Computer Motion, Goleta, CA, USA) and Da Vinci (Intuitive Surgical, Sunnyvale, CA, USA). Both systems are based on the same principle of a console providing display and command (master arm), combined to a control unit and a three-arm surgical manipulator (slave arm). The main difference between the 2 systems is the number of degrees of freedom (DOF): 7 for Da Vinci arms plus the grip motion versus 5 for Zeus. Many other reported projects led to prototypes, but only few of them were applied in surgical practice. This may be explained not only by economic factors, but also by pejorative practical aspects for a routine use (setup, maintenance, cumbersomeness). To overcome these obstacles, a close partnership between engineers and surgeons must be established and robot specifications should be thoroughly discussed between these partners. The trade-off between the specificity of the robot and its capability to be used in a relatively large range of situations should be carefully determined in the specifications. Microsurgery concerns many surgical fields such as ophthalmology, neurosurgery, and reconstructive surgery. Based on the approach, 2 procedure types can be distinguished: open (ophthalmology, plastic surgery) and keyhole (otology, neurosurgery). Difficulties of the latter are related to the narrow and confined access to the target area. The middle ear microsurgery is performed in a relatively deep and small cavity on small and fragile structures such as the tympanic membrane and the ossicles. It is conducted under an operating microscope. The vision is the predominant sensory feedback. Surgeon's hands can easily limit the field of vision, and their accuracy and tremor can hamper the procedure. The surgical exposure is often impaired because the axis of visualization is collinear to the instruments [6]. Existing prototypes dedicated to otology such as the Steady Hand [7] and MMS-II [8] did not take into account 2 major requirements in their specifications which are the preservation of the visual field and the simultaneous use of multiple tools. Our objective was to develop a robotic system to assist otologists in performing middle ear microsurgery under operative microscope. Our project was based on a partnership between an industrial in medical devices, otologists, and researchers in robotics. This work will present the design method to address several issues related to the keyhole microsurgery.

## 2. Rationale

The robot specifications are closely related to the ear anatomy, the mechanical characteristics of the middle ear, and the possible procedures which are currently performed on this organ.

### 2.1. Surgical Environment

**Figure 1:**
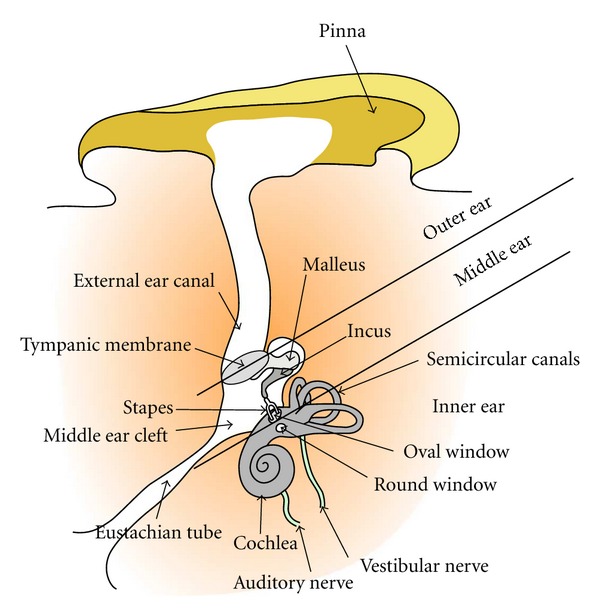
Schematics of a right human ear in operative position.

The auditory organ is composed of 3 functional parts (Figure 1): outer, middle, and inner ears. The outer ear (i.e., pinna and external ear canal) collects and directs the sound waves to the surface of the tympanic membrane. The middle ear composed of the tympanic membrane, 3 ossicles (i.e., malleus, incus, stapes), the middle ear cleft, and the mastoid air cells amplifies and delivers the acoustic energy to the inner ear via the oval window. The amplification is insured by the leverage effect between the malleus and the stapes and the tympanic membrane/round window surface ratio. The inner ear (cochlea) transforms the sound into electrical signals conveyed to the brain by the auditory nerve.

### 2.2. Middle Ear Surgical Procedures

**Figure 2:**
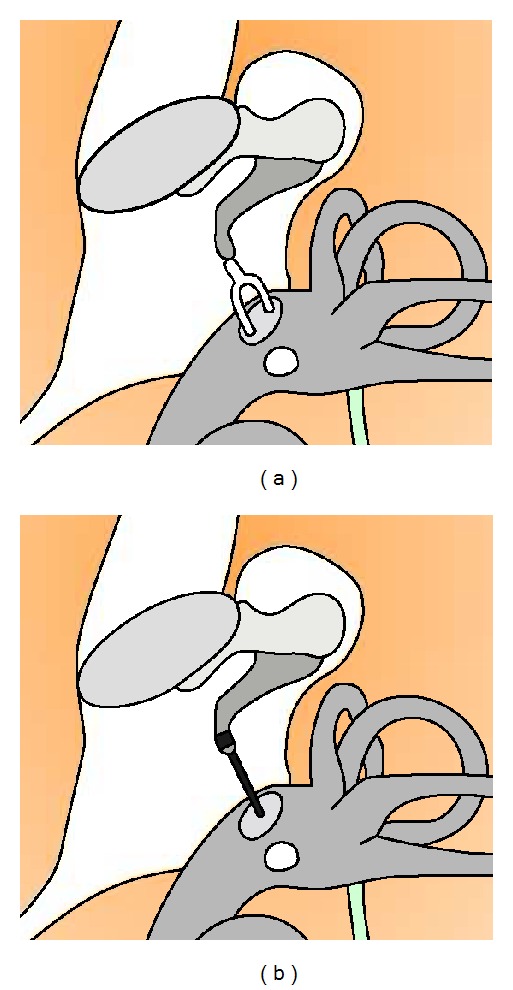
Schematics of a human middle ear representing the workspace in otosclerosis surgery before (a) and after (b) removal of the stapedial arch, perforation of stapedial footplate (platinotomy), and placement of a prosthetic piston (black) between the incus and the platinotomy.

Diseases involving the external and middle ears lead to a conductive hearing loss and can be rehabilitated by microsurgery. Sensorineural hearing loss secondary to cochlear lesions, the auditory pathways, or the central nervous system may also be rehabilitated by surgically implanted devices. Modern middle ear microsurgery was initiated in the fifties after the introduction of the operative microscope and is today widely practiced. Our study focused on middle ear procedures for the rehabilitation of the conductive hearing loss which represent the majority of otological procedures. These operations consist of tympanic membrane grafts or ossicular chain reconstruction. Among these procedures, otosclerosis surgery is the most delicate since it requires the opening of the inner ear space [9]. It was chosen as a model for the development of our robotic system. Otosclerosis is a bone dystrophy leading to a stapes fixation, impairing its vibration and the sound transmission to the inner ear (Figure 1). Otosclerosis surgery consists of removing the stapedial arch, perforating the stapedial footplate (platinotomy), and placing a prosthetic piston between the incus and the platinotomy [10] (Figure 2). The procedure is highly reproducible. However, it has an inherent risk of cochlear lesion by inner ear trauma or inflammation related to the manipulation of the stapes.

The procedure is performed through a narrow operation field offered by a speculum placed in the external auditory canal. The exposure path is tunnel-shaped, and the surgeon has to manipulate the tools nearly parallel to the axis of his vision [6]. To expose the stapes, it is usually necessary to remove the posterior-superior bony rim of the external ear canal in its medial part by a curette or a drill.

During an ossiculoplasty, the interaction forces between the tool and the ossicular chain are low (<1 N) and their slight variation cannot be easily perceived by the surgeon [11]. The success of the procedure is dependent on the surgeon's dexterity and experience [12]. Partial or complete restoration of conductive hearing loss is observed in up to 90% of cases [9]. In case of complication, a profound and irreversible sensorineural hearing loss may occur (0.2 to 3% of the cases) [13]. Taking into account the surgical challenge, the potential hearing improvement, and the necessity for precision, a robotic assistance can potentially improve procedure safety and precision.

## 3. State of The Art

### 3.1. Robotic in Middle Ear Surgery

Only few works are reported on robot-based middle ear surgery. These reports focus on only one task in the procedure such as the stapedial fenestration or prosthesis crimping around the incus. Baker et al. reported the requirements for a robot-based drilling tool to fenestrate the stapedial footplate [14]. Another similar robot was designed and assessed for an atraumatic cochleostomy (cochlear opening) [15]. In these devices, force, torque, and motion were monitored to detect the bone breakthrough and to stop the drill. Another co-manipulated robot, initially designed for ophthalmologic surgery and designated as “the steady hand,” was used to perform a stapedial fenestration with a micropick [7]. With this robot, the cumulative force applied to the stapes footplate was reduced in comparison to the standard manual technique by 31 N [11] (−58%) and the maximum force by 1.08 N (−17%). The maximum force for crimping the prosthesis was also diminished [16]. Maier et al. designed a teleoperated micromanipulator with 4 DOF called MMS-II [8]. In their project, they addressed sterilization issues and implemented the use of standard instruments. However, their system had a very small workspace (20 mm) unadapted to the length of the external auditory canal and did not provide a sufficient number of DOF to perform all middle ear procedures. To our best knowledge, no robotic device fulfills all requirements necessary to perform a complete procedure in middle ear surgery. The objective of this work was to design and evaluate a robot capable of performing all tasks in middle ear surgery procedures.

### 3.2. Design Process

Several works have already described the design process in various robotic fields. All these works follow the same process beginning by design specifications and followed by the choice of kinematics, the definition of optimization criteria, the optimization, the manufacture of a prototype, and finally the evaluation. This process addresses specific issues related to the surgical specialty. Technical options to overcome mechanical limitations of the human hand and the ergonomic options underlie the entire design process. This process has been applied to robots for laparoscopic surgery and microsurgery and has yielded several prototypes [16–21].

## 4. Design Requirements

In order to identify all design requirements relative to middle ear microsurgery, it was crucial to study and characterize the workspace, the force capability, and the accuracy [22, 23]. The workspace allowed choosing the adequate kinematic. The selection of actuators was based on force capability and the accuracy.

### 4.1. Extracorporeal Workspace

**Figure 3:**
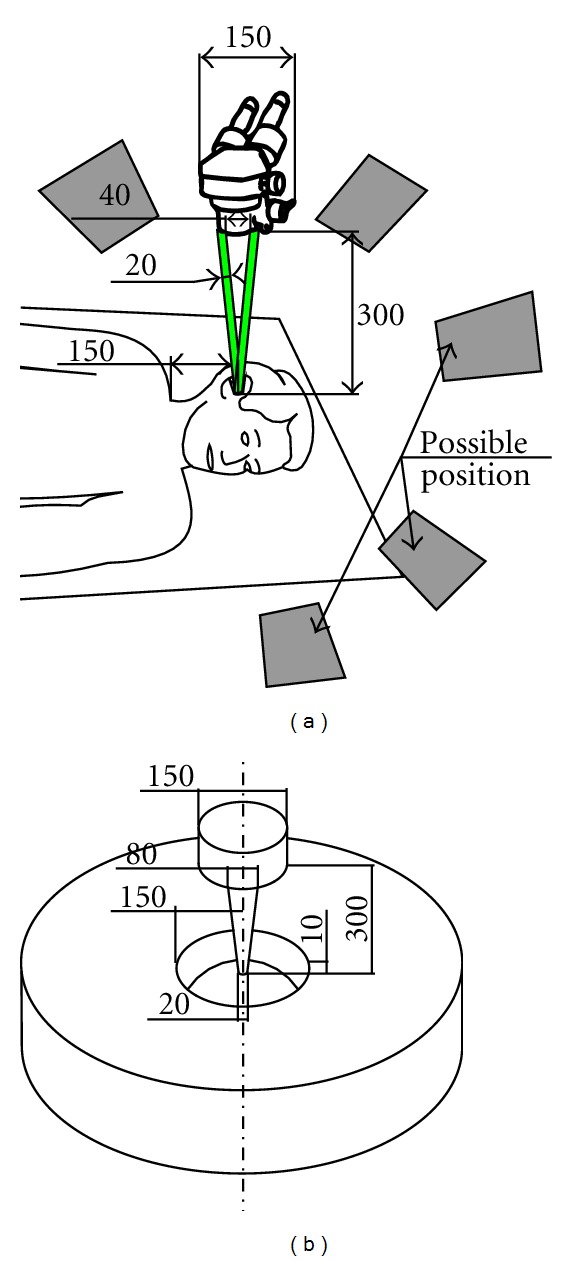
Extracorporeal space assessment (dimensions in mm). Gray surfaces represent possible positions of the robot base. Green surfaces represent the field of vision. The 360° revolution of the planar model (a) led to a 3D model of the extracorporeal space (b) around which the robot and the microscope could be placed freely.

The objective of this part of the study was to prevent collision between the robot and the extracorporeal workspace and to preserve the visibility of the surgical field through the operative microscope. In ear surgery, the microscope is routinely used to provide a magnified stereoscopic vision and lighting of the confined surgical field. It also provides a video output for shared vision and documentation. It is combined to a laser system for vaporization or cutting purposes. In order to take into account the dimensional limitations imposed by extracorporeal obstacles such as the microscope, a “patient-microscope-visual field”*‌* group was modeled. In this assessment, only the exterior shape and dimensions of the microscope were relevant. The relative positions of all structures depend on the microscope's focal distance. The commonly used focal length for ear surgery is in the range of 250 to 300 mm. To maximize the available space, this distance was set at 300 mm. The distance between the speculum axis and the upper limit of the thorax was set at 150 mm. The visual field consisted of 2 cylinders representing the 2 eyepieces and intersecting at the focal point. They were 40 mm apart and had a 20 mm diameter. The microscope was 150 mm wide and was coaxial with the speculum placed in the ear canal. The maximum height between the patient's chest and the pinna was set at 10 mm. The visual field diameter at the focal point was 20 mm (Figure 3(a)). All identified dimensions were expressed in relation to the operated ear considered as the reference. The axial orientation of the microscope and the robot placement could vary depending on patient's morphology and variations of the surgical procedure. Consequently, we enabled the robot's base and the microscope to move freely around the patient's head by transforming our planar model into a 3D space via a 360° revolution around the view axis (Figure 3(b)).

### 4.2. Intracorporeal Workspace

**Figure 4:**
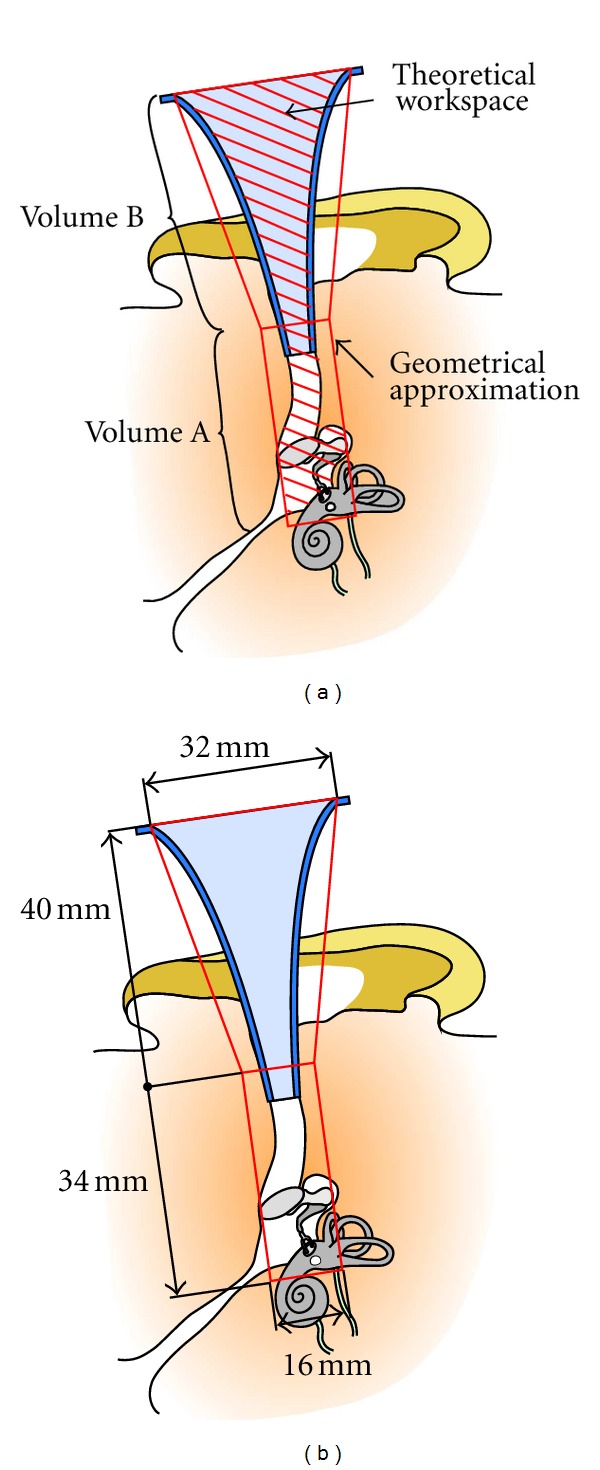
Intracorporeal workspace: schematics of a right human ear in the operating position are represented. The theoretical intracorporeal workspace (hatched surface) was maximized, and a geometrical approximation (cylinder + truncated cone) was obtained (a). Maximal dimensions were estimated based on CT scan data (b).

The objective of this part was to model the workspace for surgical instruments. This space included all possible instrument positions and orientations in middle ear procedures. The theoretical workspace is represented as the hatched surface in Figure 4(a). Otosclerosis surgery which was chosen as the reference procedure for specifications is conventionally carried out through the ear canal with an ear speculum. The dimensions of the outer and middle ears and the speculum were therefore essential. The bloc of auditory canal plus the visible part of the middle ear cleft was approximated by a cylinder (volume A) and the speculum by a truncated cone (volume B). There is a significant inter individual variation of ear morphology. For this reason, the calculation of the volume A was based on anatomical data from CT scans. The study was conducted on preoperative CT scans of 12 patients by using 3-dimensional multiplanar reconstructions. The highest values were taken into account for the workspace dimensions. The maximum measured height was 34 mm, and the maximum diameter was 16 mm. The standard speculum model used for middle ear surgery had a diameter ranging from of 6 mm at the narrower end to 32 mm at the larger end and a height of 40 mm (Figure 4(b)).

The robot's workspace was defined as a reachable space in position consisting of volumes A and B and a reachable space in orientation consisting of all possible orientations when the tool tip is in volume A. Volume B was not a part of the surgical field but merely an approach space. Tool orientations in this field were not taken into account, and the extreme orientations determined for volume A were simply applied to volume B.

### 4.3. Force Capacity

**Figure 5:**
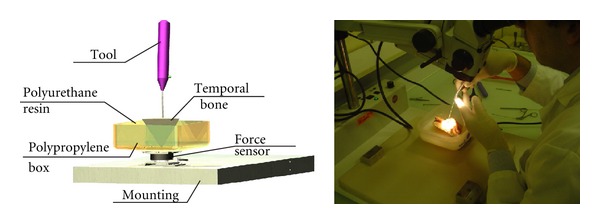
Test bench for force measurements: this bench allowed measurements during a manual procedure in realistic situation.

Forces necessary to perform all steps of the surgical procedure were specified. Few data were available in the literature [11, 16, 24]. For this purpose, a specific test bench was designed allowing dynamic force measurements while surgeons perform gestures in a realistic environment (Figure 5). A fresh temporal bone was immobilized in a polypropylene container with a polyurethane resin. A 6-axis force sensor (ATI nano 43, ATI Industrial Automation, Apex, NC, USA) was attached under the container and was connected to a PC. An in-house software insured a real-time force measurement and recording (RTAI: real-time application interface for Linux). Forces applied on the temporal bone during different tasks (i.e., mastoidectomy, external auditory canal bone drilling, and stapedial footplate fenestration) were measured by the force sensor. Two otologic surgeons performed the tasks on 8 human temporal bone specimens. Each gesture was measured 10 times. Force was plotted against time, and the peak force was recorded. For each task, minimal and maximal forces were noted. The highest value was recorded for the external auditory canal bone drilling and was 4.25 N [22]. Force capability was maximized and set at 5 N for the robot specification.

### 4.4. Accuracy

**Figure 6:**
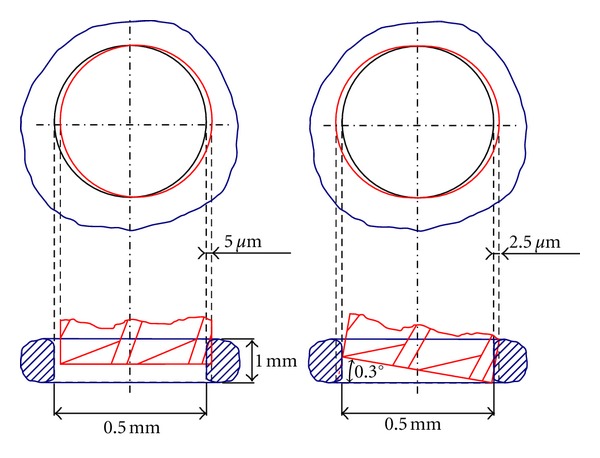
Geometric analysis of accurany in a platinotomy. One percent error of platinotomy corresponded to 5 *μ*m and 0.3° for linear and angular resolutions, respectively.

The goal in this part of the project was to evaluate the linear and angular accuracy needed to perform a middle ear procedure. A limited accuracy would make the robot useless for the microsurgery while an excessive accuracy would increase the cost. The most delicate and important gesture is the stapedial footplate fenestration that directly affects the functional outcome and the risk of otosclerosis surgery. This round fenestration typically measures 0.5 mm in diameter. A simple geometric analysis of the platinotomy was performed assuming a stapes footplate thickness of 1 mm [25]. An error of position or form up to 1% of the drilling diameter was considered as acceptable. This analysis led to a linear resolution of 5 *μ*m and an angular resolution of 0.3° (Figure 6). 

The robotic system was designed to be controlled by the surgeon using his own vision as a control loop. So the position accuracy depended only on the image quality and on the resolution of the micromanipulators. Hence, this resolution had to be less than 5 *μ*m in translation and 0.3° in rotation. The angular accuracy was easily obtained by standard rotary engines with 512-point quadrature encoders providing a resolution below 0.18°. In contrast, obtaining a 5 *μ*m linear accuracy represented a technical challenge. Indeed, very high angular resolutions can be linked to unacceptably low linear resolutions depending on the kinematics. For instance, the compact and high-quality industrial manipulator robot TX40 (Stäubli, Pfäffikon, Switzerland) was equipped with a high-resolution (0.01°) actuator on its last link. However, assuming that the tool measured 150 mm long similarly to our robot, the linear resolution at the tool tip was low (>25 *μ*m).

### 4.5. Conclusion

For middle ear surgery, the robotic system which is placed between the patient and the microscope should avoid collisions (Figure 3) and preserve the visual field through the microscope in all positions. It should have at least 6 DOF and be able to reach the entire workspace with its tool both in position and orientation (Figure 4). It should also be capable of producing a 5 N force at the tool tip in all space directions with a linear resolution below 5 *μ*m and an angular resolution below 0.3°. After setting the above specifications, the kinematic was selected and optimized.

## 5. Kinematic Structure Selection

### 5.1. Overview of Preselected Kinematic Structures

**Figure 7:**
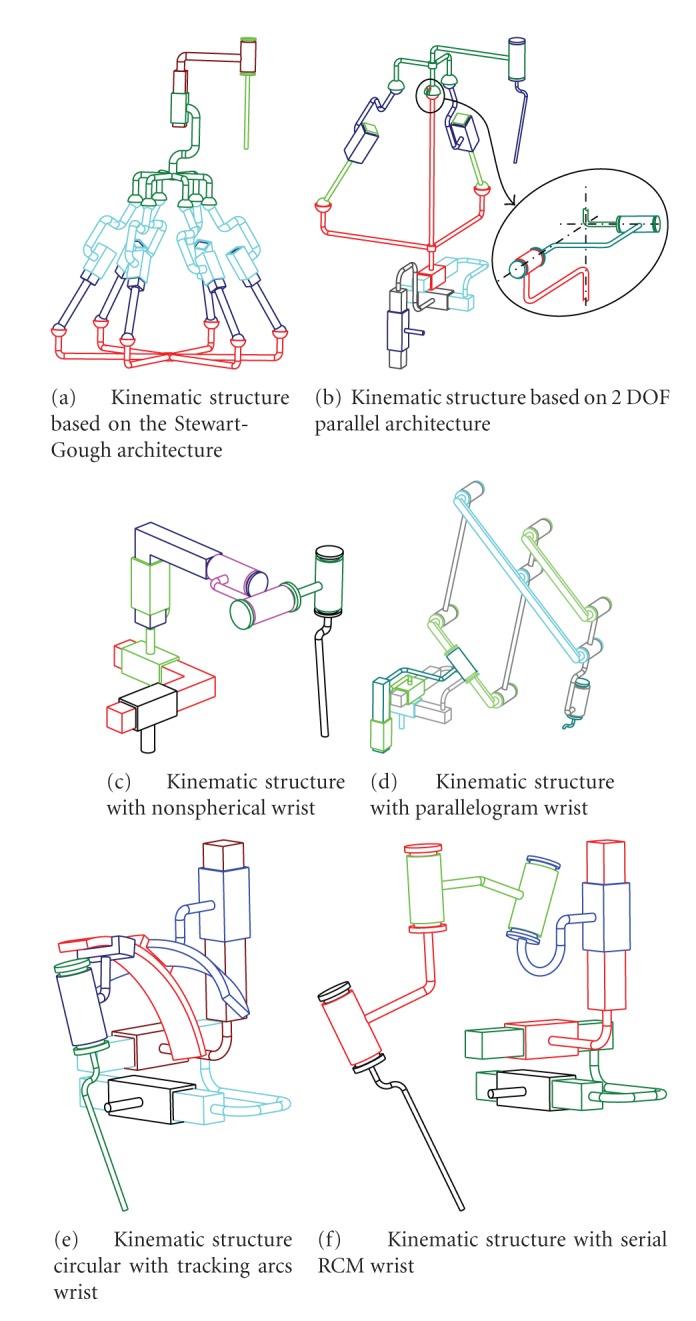
Kinematic structure selection.

The goal of this part was to study and to select the most suitable kinematic for middle ear surgery. Many researchers reported on the kinematic architecture in surgical robotics by compiling publications on the state of the art [26, 27]. Initially, assistance robotic systems in the medical field were based on kinematics commonly used in industry. Today, many systems are based on dedicated kinematic architectures. Medical and surgical robots are principally based on anthropomorphic, selective compliant assembly robot arm (SCARA), parallel and Cartesian architectures, often including a serial wrist. The serial wrist may be nonspherical, spherical, or with a remote center of motion (RCM). SCARA and anthropomorphic kinematics have a relatively large workspace center around their base. This characteristic is useless for middle ear procedures which are conducted in a very narrow and off-centered workspace. However, these architectures could be potentially applied to a robot-carrier combined to an otologic micromanipulator. Parallel kinematics have the advantage of a high structure rigidity, speed, and accuracy over serial structures. The rigidity allows displacing the actuators from a distal location to the robot base in order to reduce the volume at the distal part of the arm and the visual impairment [7, 28, 29]. However, these robots have the major drawback of occupying a large volume for a limited workspace. A serial wrist is often included in the kinematic structures of surgical robots. It represents a fine structure consisting of revolute serial links allowing a simple calculation of their model [30]. Kinematic structures with RCM are present in both minimally invasive surgery and microsurgery [27]. This kind of structure is suitable for manipulating long tools by a proximal prehension and orientating the surgical instruments without changing their distal position. This characteristic is crucial for middle ear surgery considering the localization and the shape of the workspace. This type of architecture may be based on circular tracking arcs. Mainly used in eye surgery [31–33], it provides a high angular resolution (on circular tracking arc segments) and is adapted to the human head anatomy. Kinematic structures with RCM may also be based on a double parallelogram combining the advantages of both parallel and RCM structures [17, 21]. Our specifications imposed a high linear resolution favoring the choice of prismatic links. The shape of the internal workspace necessitated large displacements along the tool axis both in orientation and translation. For this reason, kinematic structures with terminal rotary link and providing a self-rotation of the tool were privileged. The anatomical characteristics of the workspace also imposed rotations with a center close to the distal part of the instrument. This requirement oriented the choice toward a kinematic with RCM. Finally, it was necessary to limit the global size of the robot and to preserve the visual field, and this argument was in favor of a serial structure. Based on these remarks and on the workspace properties, 6 candidate structures were preselected (Figure 7). These kinematic structures were serial, parallel, or mixed and had at least 6 DOF, as required in the specifications.

### 5.2. Kinematic Structure Selection Process

Kinematic structures depicted in Figures 7(a), 7(b), and 7(c) had a natural rotation center distant from the tool tip, but the natural rotation center had to be displaced to perform a pure rotation around the tip. Surgical tools measure 150 mm in length in average. Rotating the tool around its tip with a 25° angle needed a displacement of the center of approximately 65 mm. This value was close to the height of the workspace. In order to limit the necessary linear movement, kinematic structures with a center of motion close to the tool tip were retained and other preselected structures (Figures 7(a), 7(b), and 7(c)) were rejected.

All retained candidates had 6 DOF and a RCM wrist allowing coincidence between the tool tip and the center of motion (Figures 7(d), 7(e), and 7(f)). The first kinematic (Figure 7(d)) was composed of a double parallelogram mechanism. It allowed placing most of the actuators close to the robot base and to ensure high rigidity. However, the tool length (150 mm) imposed a large parallelogram incompatible with the microscope focal length. The second candidate (Figure 7(e)) was based on circular tracking arc mechanisms. On one hand, circular arcs are appropriate for surgery around the head. On the other hand, placement of two such robots under the microscope without collision of the two robotic wrists and with a preserved visual field was difficult to envisage. The last solution (Figure 7(f)) was composed of three prismatic links followed by a serial RCM wrist which has its rotation center in coincidence with the tool tip. This structure did not have the drawbacks of the 2 previous architectures in terms of size and complexity and was finally selected as the suitable solution.

## 6. Optimization Process

Before beginning the optimization process, it was necessary to parameterize the robot's kinematic and to define the optimization criteria. These criteria were defined according to the specifications on the internal workspace accessibility and the preservation of the visual field.

### 6.1. Parameterization of the Kinematics

**Figure 8:**
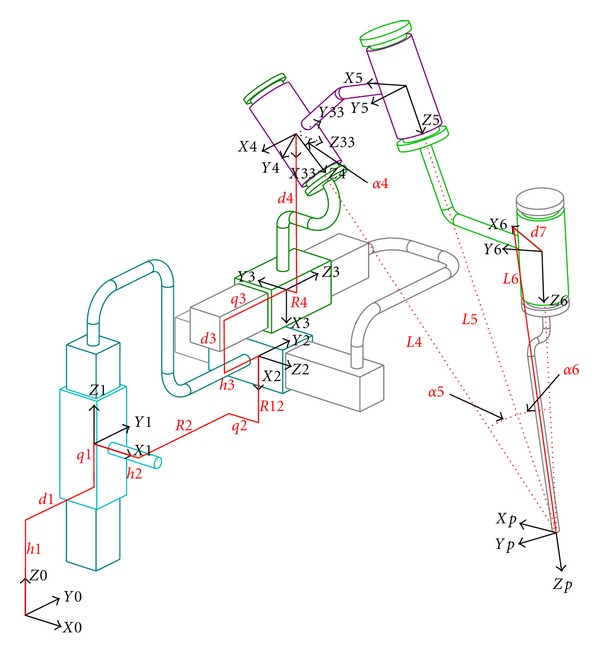
Kinematic structure parameterization.

The selected structure was composed of 3 linear perpendicular links placed in series. Its movements were provided by 3 independent motorized linear stages. Three rotary links in series followed in the kinematic chain. To obtain a RCM, these 3 rotary links had their axes intersecting at a point coinciding with the tool tip. The last rotary link axis was different from the tool axis in order to increase possible configurations and to maximize the visual field under the operating microscope. Motions of these links were insured by 3 independent rotary motors.  Figure 8 shows the kinematic scheme with its parameterization. Each body “*i*” is assigned with a reference frame. Its *z*-axis is set as the *z*-axis of the link “*i*”. Dimensional parameters “*hi*, *Ri*, *qi*, *di*, *α*i and *Li*” describe the path from a reference frame to another. Note that this parameterization does not exactly follow the Denavit-Hartenberg (DH) conventions commonly used in robotics. This variation facilitates interpretation of different values. In particular, it can establish a link with technological information such as linear stage size or the distance between the position of the RCM and the revolute link center. Moreover, this parameterization allows calculating the homogeneous transformation matrices characterizing the direct geometric and kinematic models of our serial chain. Model parameters can be divided in two types:

- 6 joint parameters, *q*1, *q*2, *q*3 3 translations for the cross table and *q*4, *q*5, *q*6 (not shown in Figure 8) for the 3 rotations,
- 16 dimensional parameters divided into 4 distinct groups.

### 6.2. Parameter Analysis

**Figure 9:**
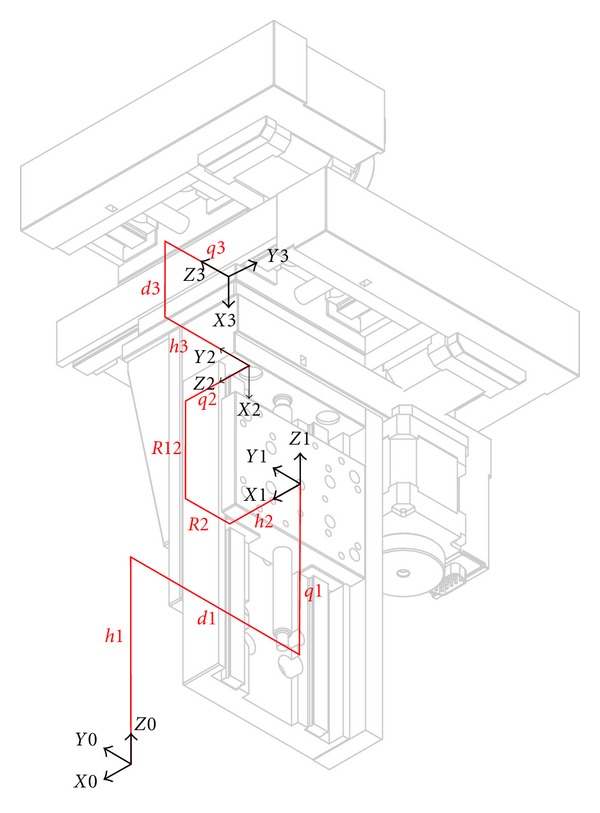
The optimal cross table.

 Group 1 consisted of parameters *d*1 and *h*1. These parameters positioned the cross table base in the area of the frame reference *ℛ*_0_. They were defined in such a way that the cross table is placed far from the patient's upper thorax and the microscope. Group 2 included *h*2, *h*3, *d*3, *R*2, and *R*12 which characterize the respective positions of the linear stages. The values of these parameters were deduced from the sizes of the linear stages and their combination led to a cross table as compact as possible (Figure 9).

Group 3 composed of *d*4, *R*4, and *L*4 positioned the RCM wrist and the tool tip according to the cross table. Group 4 included *α*4, *α*5, *α*6, *L*5, *L*6, and *d*7 and described the arrangement of rotary links in the RCM wrist. The parameters *α*4, *α*5, *α*6, *L*5, and *L*6 characterized the robot wrist size, *d*7 the distance between the last link axis and the tool axis on which depended the visual field preservation.

### 6.3. Exploration Area Definition

**Figure 10:**
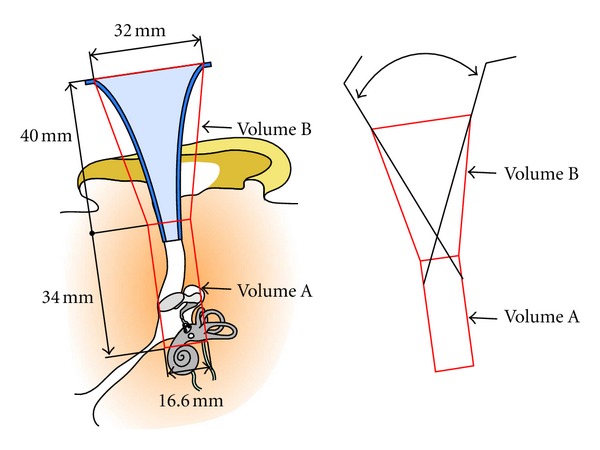
Extreme tool orientations according to the workspace.

To define the optimal values, an exploration area for the group 4 (wrist size) was first defined.  Table 1 summarizes this exploration area by providing the range of each parameter and the exploration step size, similar to the method employed by Lum et al. [34]. Extreme values of parameter *L*5 were chosen to limit the wrist size in respect to the extracorporeal environment. The parameter L6 which depended on the tool length ranged between 130 mm (the conventional tool length can reach a minimum of 100 mm) and 180 mm to limit size and minimize the collision risk. The minimum and maximum angles *α*4, *α*5, and *α*6 were chosen considering the extreme tool orientations imposed by the workspace (Figure 10).

The range of parameter *d*7 was based on the expected mechanical link size and the visual field diameter. Finally, group 3 parameters were deduced from the values of group 4 parameters. For a given set of values for the parameters in group 4, group 3 parameters were calculated considering that the tool tip (RCM) was located in the center of the volume A upper surface (Figure 10), and that the joint values *q*1, *q*2, *q*3 placed the linear stages at midstroke. 

An area comprising 138,240 sets of possible values was defined (Table 1). The goal was to optimize the parameters as a function of design specifications and to find the optimal set of parameters for the middle ear robot. Each set of parameters represented a candidate. Each candidate was evaluated for multiple criteria and compared with all others [34].

### 6.4. Optimization Criteria

#### 6.4.1. Candidate Evaluation

**Figure 11:**
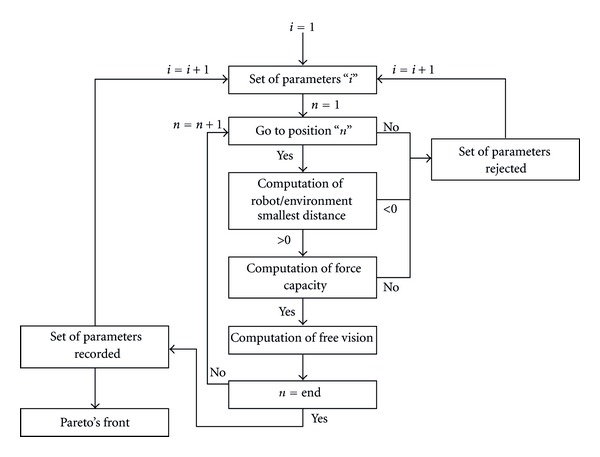
Algorithm for candidate selection among all kinematic solutions according to specifications.

First, each candidate had to reach all positions and orientations of the workspace. In addition, the candidate had to avoid collision with extracorporeal environment, to produce a 5 N force in all directions, and to preserve the visual field in all configurations. To assess candidate, an in-house software was developed in Matlab. This software included several functions of Toolbox Robotics [35] and Arboris Library [36]. It was based on an algorithm shown on Figure 11. For one set of parameters, the robot capability to follow a representative 6D path was computed. For each configuration, the candidate had to fulfill the non-collision criteria. Thus, the computation of the distance between the candidate and extracorporeal environment had to be performed and recorded. Subsequently, the 5 N force ability of the candidate was verified. Finally, the visual field preservation was evaluated: the nonobstructed area corresponding to the free vision was computed and recorded. If the candidate was not able to reach a configuration of the path, if it was in collision with the extracorporeal environment, or if its force ability below the 5 N limit, the candidate was rejected. When a candidate reached all the configurations of the path and respected all the criteria, it was preselected and scored. Its score was based on its smallest recorded distance to the environment and on the average percentage of free vision in different configurations.

#### 6.4.2. Definition of Representative Path

**Figure 12:**
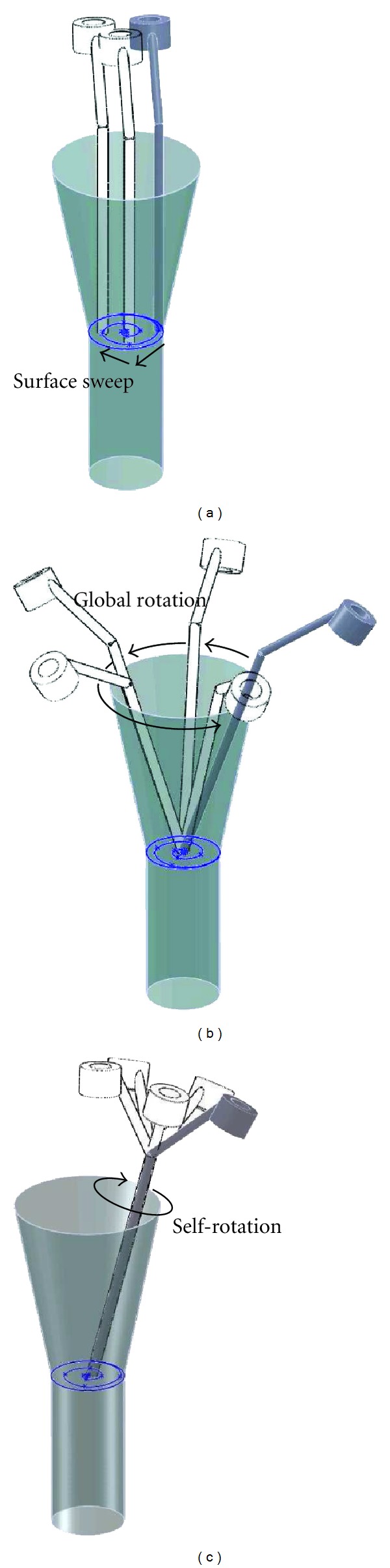
Tool trajectory in optimization process. (a) Surface sweep, (b) rotation with a fixed tool tip, (c) rotation around tool axis.

Different tool configurations with maximum angles, representing the most complex tool path, were assessed. Such a representative 6D path was defined and is illustrated in Figure 12. The tool tip moved from its starting location to the operating area within the workspace in 3 configurations. Then the tool tip swept the upper surface of the volume A in 30 configurations (Figure 12(a)). This movement was composed of a circular path which followed the upper extremity surface of the volume A (in 17 configurations) and of a spiral shape path which ended on the upper surface center of the volume A (in 13 configurations). For each configuration of this movement, the lateral part of the tool had to follow the upper surface of the volume B in 9 steps (Figure 12(b)). Then, for each of these configurations, the tool had to be able to turn around its axis in 9 steps (Figure 12(c)). This 6D trajectory comprised 2433 configurations. 

It should be noted that this path explored only the upper surface of the volume A. The accessibility to other points in the workspace is insured by the stroke of the linear stages. Moreover, the changes of tool axis orientations were greater when the tool tip was on the upper surface of volume A in comparison to the tool tip below this surface but in the same volume. The volume B was an access road to the operating area, and for this reason the reachable orientations were not tested in this area. The selected kinematic structures permitted to uncouple the linear and angular motions of the tool, so the reachable orientations were available in all the workspace including the volume B.

### 6.5. Evaluation of Path Pursuit

The implemented algorithm evaluated the reachability of each path configuration. A configuration of the reference trajectory was reached when a corresponding solution in terms of joint parameters was found. Let ${\tilde{T}}_{0}^{p}$ be the homogeneous transformation matrix of the target configuration. Let *T*_0_^*p*^ be the homogeneous transformation matrix for the candidate current configuration:

(1) $$\begin{array}{c} {\tilde{T}}_{0}^{p}=\left[\begin{array}{cccc} {\tilde{n}}_{x} & {\tilde{o}}_{x} & {\tilde{a}}_{x} & {\tilde{p}}_{x}\\ {\tilde{n}}_{y} & {\tilde{o}}_{y} & {\tilde{a}}_{y} & {\tilde{p}}_{y}\\ {\tilde{n}}_{z} & {\tilde{o}}_{z} & {\tilde{a}}_{z} & {\tilde{p}}_{z}\\ 0 & 0 & 0 & 1 \end{array}\right],\;\;T_{0}^{p}=\left[\begin{array}{cccc} n_{x} & o_{x} & a_{x} & p_{x}\\ n_{y} & o_{y} & a_{y} & p_{y}\\ n_{z} & o_{z} & a_{z} & p_{z}\\ 0 & 0 & 0 & 1 \end{array}\right]. \end{array}$$

The joint parameters matching with the homogeneous matrix ${\tilde{T}}_{0}^{p}$ had to be determined by using one of the closed-loop inverse kinematics methods. Hence, the differential vector *d*_*x*_ materializing the difference between the required configuration and the current configuration was computed as follows [37, 38]:

(2) $$\begin{array}{c} d_{x}=\left[\begin{array}{c} \vec{d_{p}}\\ \vec{d_{\theta }} \end{array}\right]=\left[\begin{array}{c} \vec{\tilde{p}}-\vec{p}\\ \frac{1}{2}\left(\vec{\tilde{n}}\wedge \vec{n}+\vec{\tilde{o}}\wedge \vec{o}+\vec{\tilde{a}}\wedge \vec{a}\right) \end{array}\right]. \end{array}$$

Then this differential vector was projected in the robot joint space to obtain a corrected articular vector *d*_*q*_:

(3) dq=J(q)−1dx.

Starting from the current joint configuration *q*, the new joint configuration $\bar{q}$ was calculated by applying a proportional gain *λ*:

(4) q¯=q+λdq.

The joint configuration $\bar{q}$ was updated to the current joint configuration and the process continued until the displacement *d*_*x*_ was complete:

(5) ||dp→||<εp,  εp=0.1 μm,||dθ→||<εθ,  εθ=0.1 e−6°,

with *ε*_*p*_ and *ε*_*θ*_ the thresholds for convergence in position and orientation, respectively. If the configuration was not reached in 15 iterations with *λ* = 1, the candidate was rejected.

### 6.6. Evaluation of Distance to Obstacles

**Figure 13:**
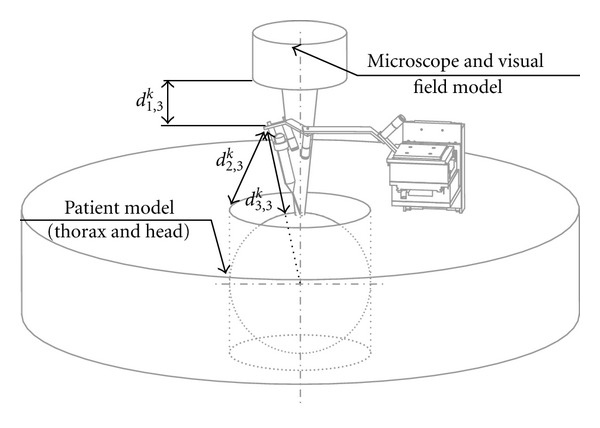
Model of robot distances to the obstacles.

The second tested criterion was the distance between the robot and the extracorporeal environment. It was essential for the robot to reach the entire workspace without colliding with the microscope, the patient's head, or the upper thorax. For each path configuration (index *k*), the distance *d*_*i*,*j*_^*k*^ between each link center (index *j*) of the candidate and each volume of the external environment (index *i*) was calculated (Figure 13). As the robot arm had 3 links and the external environment was composed of 3 volumes, 9 distances were computed.

It was not necessary to check if the cross table collided with the environment since it was initially positioned away from the obstacles. If a computed distance was negative, it meant that there was a collision and the candidate was rejected by the algorithm. If instead all distances were positive, they were recorded and the candidate passed to the next path configuration. At the end of the path, the lowest value among all distances yielded the initial score *S*_*o*_ for obstacle avoidance:

(6) $$\begin{array}{c} S_{o}=\underset{i,j,k}{\min}\left(d_{i,j}^{k}\right)\;\text{with}\{\begin{array}{c} i=1,2,3\;\;\text{obstacles}\\ j=1,2,3\;\;\text{links}\\ k=1,\ldots ,2433\;\;\text{configurations}. \end{array} \end{array}$$

### 6.7. Evaluation of Force Capacity

**Figure 14:**
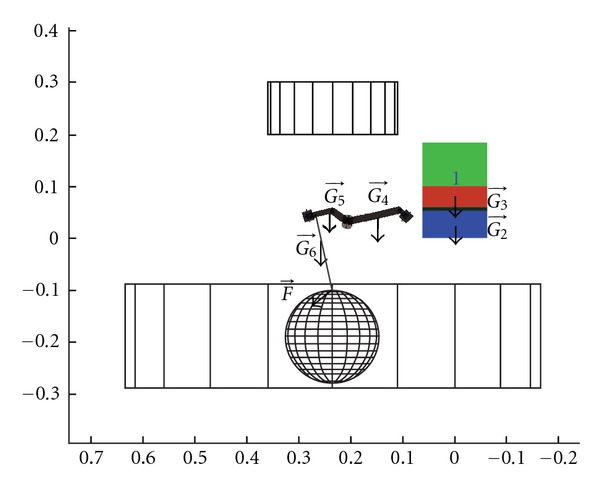
Illustration of the static computation.

The robot had to be able to provide a 5 N force in all space directions. The load capacities of robot actuators were known. This allowed determining if these load capacities were sufficient to bear the robot weight and to provide the tool tip with a 5 N force in all directions for each configuration of the evaluation path. This computation was based on the static model, on the actuator characteristics, and on the tool weight (Figure 14):

- *τ* = *J*(*q*)^*T*^ *F* + *G*(*q*),
- *τ*: the vector (6 × 1) representing the force provided by the actuators,
- *J*(*q*): the Jacobian matrix of the candidate,
- *F*: the vector (6 × 1) representing the force on the effector,
- *G*(*q*): the vector (6 × 1) representing the weight of each body.

For this computation, the tool weight was set at 500 g. This weight represented the surgical drill which is the heaviest tool used in ear surgery. If the candidate was able to produce the required force in the tested configuration, the candidate passed to the next path configuration. If not, it was rejected.

### 6.8. Visual Field Preservation

**Figure 15:**
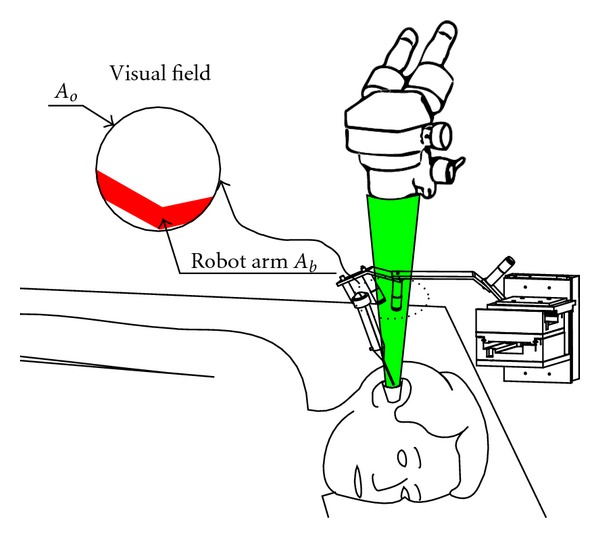
Interference of the robot arm with the visual field under microscope.

During actuation of the tool by the robot, its wrist interfered with the visual field (Figure 15) causing a partial loss of visual feedback to the surgeon. The goal was to minimize this loss. In each configuration of the evaluation path, the candidate's wrist hid a part of the visual field. The percentage of the free field was computed by subtracting the projected wrist surface (red part in Figure 15) from the entire visual field surface (a circle). If the candidate reached all the configurations of the evaluation path, the average percentage of the free field was calculated as the second score *S*_*v*_:

(7) $$\begin{array}{c} S_{v}=\frac{100}{n}\sum_{1}^{n}\frac{A_{o}-A_{b}}{A_{o}}\;\;\text{with}\;\;n=\text{configurations}\;\;\text{number}. \end{array}$$

## 7. Optimization Results

### 7.1. Overall Evaluation

**Figure 16:**
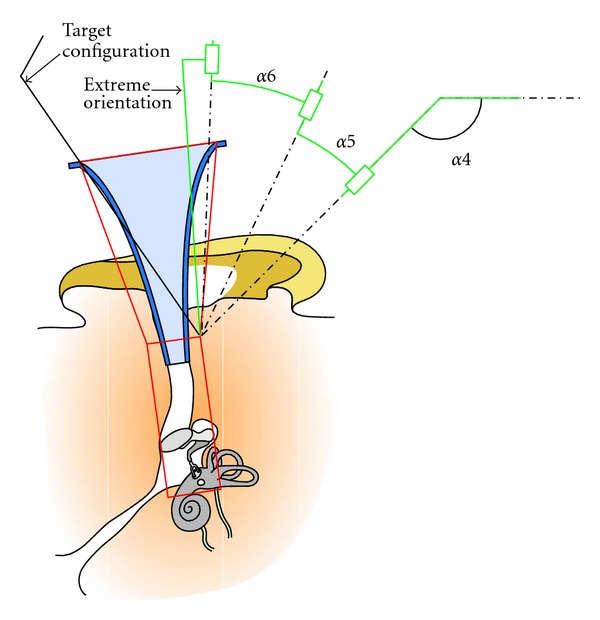
Unreachable configuration due to the wrist dimension.

The exploration zone (Table 1) was divided to 8 subdomains, and each subdomain was explored on a separate computer (PC with Intel Celeron at 1.8 GHz, with 512 MB RAM). The exploration of each subdomain lasted approximately 200 hours. Out of 138,240 evaluated candidates, 134,177 were rejected either during the initial evaluation step due to collision with the extracorporeal environment or because they were unable to reach all path configurations. In these cases, it was impossible to reach the extreme opposite orientation (Figure 16) even with a fully extended wrist (when Z4, Z5, and Z6 axes are coplanar). However, all rejected candidates respected the force criterion.

**Figure 17:**
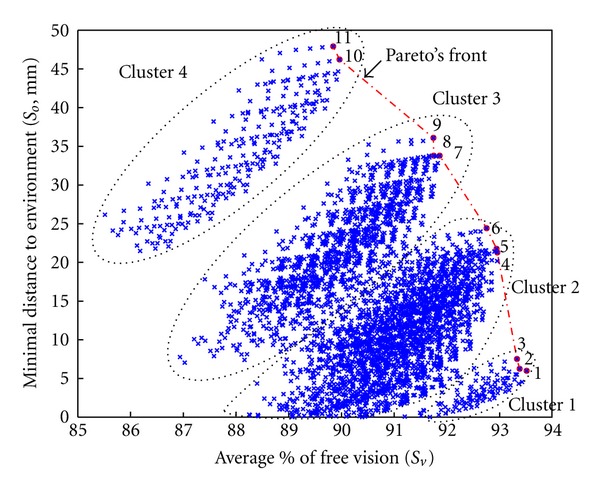
First step of result optimization. The percentage of free visual field was plotted against the minimal distance to environment (inversely related to risk of collision). Each cross represents a set of parameters which passed the overall evaluation (*n* = 4063). Pareto's front (in red) identified 11 candidates.

Only 4063 parameter sets reached all configurations of the reference path without collision with the extracorporeal environment and offered the required force capacity. To study the distribution of these successful candidates, the average percentage of free visual field along the path (in abscissa) was plotted against the minimal distance between the candidate and the environment (risk of collision in ordinate). A Pareto front was drawn in order to select the best candidate. This front helped to identify 11 candidates (Figure 17 and Table 2).

Candidates which passed the evaluation process were distributed in four clusters on the plot: (i) the smallest was situated on the lower right and included candidates 1, 2, and 3 on the Pareto's front; (ii) the largest cluster located just above the first included solutions 4, 5, and 6; (iii) the third shared several candidates with the second and included candidates 7, 8 and 9; (iv) the fourth was distant from the three others and included the two last solutions of the Pareto front (10 and 11). The minimal distance to the environment varied from 6 mm for candidate 1 to 47.9 mm for candidate 11. The average percentage of free vision ranged from 93.5% for candidate 1 to 89.8% for the 11. The latter appeared to be the best candidate since it maximized the distance to the obstacles without impairing the visual field more than other candidates. It should be noted that all 11 candidates on the Pareto front had the same values for their linear parameters (*L*5, *L*6, and *d*7). They corresponded to one of the limits of the exploration area.

### 7.2. Influence of Geometrical Parameters on Scores

**Figure 18:**
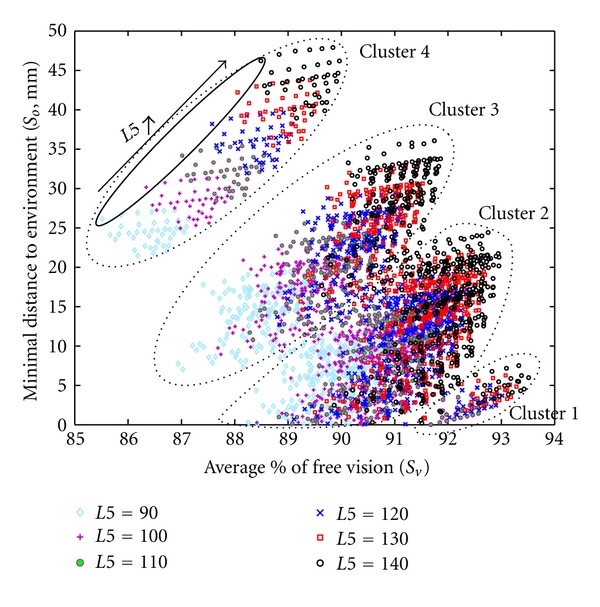
Influence of parameter *L*5 on optimization results. Each cross represents a set of parameters which passed the overall evaluation (*n* = 4063). Different symbols and colors indicate different *L*5 values in mm (insert).

A detailed analysis of the results allowed to highlight the influence of each geometrical parameter on the visual score *S*_*v*_ and on the distance score *S*_*o*_. Parameter *L*5 appeared to have a significant influence on the optimization results (Figure 18).

**Figure 19:**
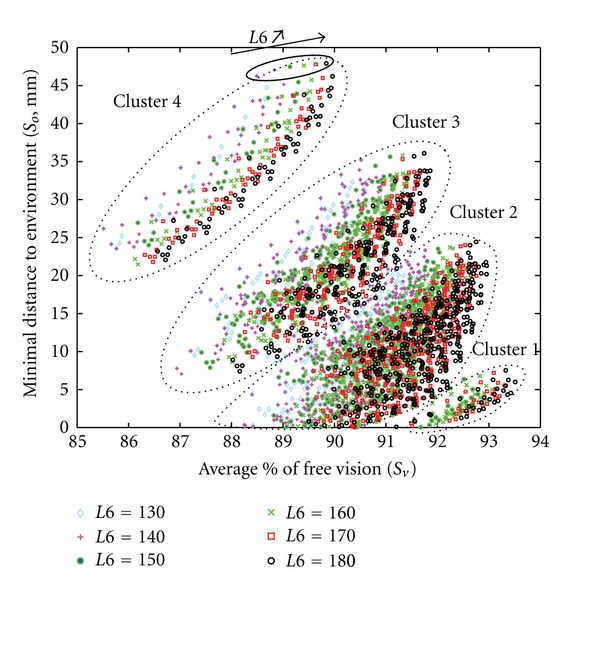
Influence of parameter *L*6 on optimization results. Each symbol represents a set of parameters which passed the overall evaluation (*n* = 4063). Different symbols and colors indicate different *L*6 values in mm (insert).

Indeed, a detailed analysis of cluster 4 revealed a horizontal stratification of candidates having the same *L*5 values (Figure 18). This stratum was sorted in an ascending order with the lowest value (90) at the bottom and the highest (140) at the top. On Figure 18 several particular solutions could also be highlighted. These solutions represented candidates having the same set of parameter values except for *L*5. These particular solutions showed that when *L*5 increased (from 90 to 140 mm), the average % of free vision and the minimum distance to obstacles also increased (from 85.5% to 88.5% and from 26 mm to 47 mm, resp.). This was the reason why all candidates indicated by the Pareto front had a maximal *L*5 value allowed by exploration area (140 mm). The evolution of the parameter *L*6 had also an influence on the candidates' scores (Figure 19).

**Figure 20:**
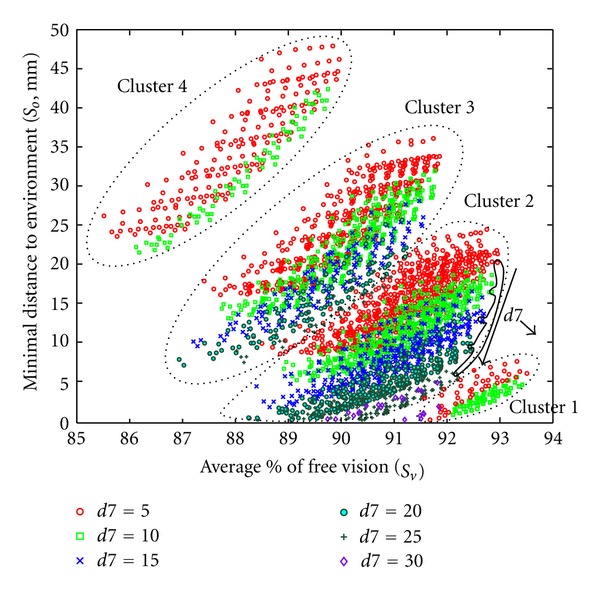
Influence of parameter *d*7 on optimization results. Different symbols and colors indicate different *d*7 values in mm (insert). Each symbol represents a set of parameters which passed the overall evaluation (*n* = 4063).

Inside cluster 4, a stratum of candidate was sorted in an ascending order, following an oblique angle and ranging from 130 at the bottom to 180 at the top. Several particular solutions are shown on Figure 19. These solutions represent candidates having the same set of parameter values except for *L*6. They show that when *L*6 increased from 130 to 180 mm, both the average percentage of free vision and the minimum distance to obstacles increased from 88.3 to 89.8% and from 26 to 47 mm, respectively. This explains that all candidates selected on the Pareto front had a maximal *L*6 value allowed by the exploration area (180 mm). The variation of the parameter *d*7 also affected the candidates' scores (Figure 20).

**Figure 21:**
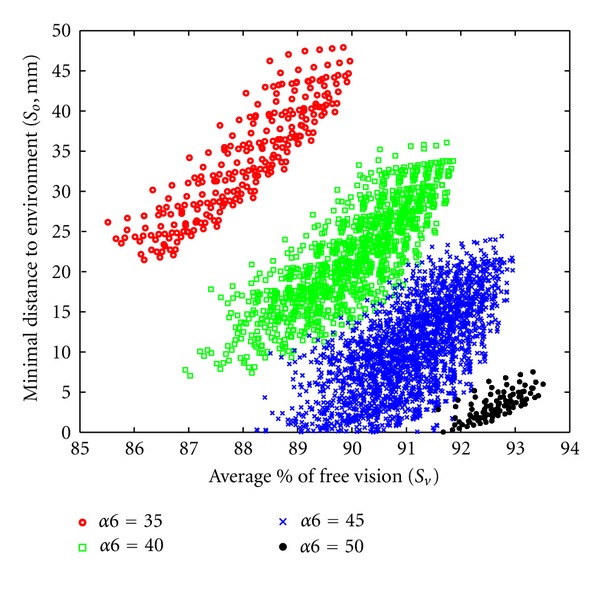
Influence of parameter *α*6 on optimization results. Different symbols and colors indicate different *α*6 values in degree (insert). Each symbol represents a set of parameters which passed the overall evaluation (*n* = 4063).

 Cluster 2 showed a stratum of candidates sorted in descending order following an oblique angle with *d*7 values ranging from 5 (top) to 30 (bottom). Several solutions could be distinguished on Figure 20 (circumscribed area). These solutions represent candidates having the same set of parameter values except for *d*7. These particular solutions showed that when *d*7 increased from 5 to 30 mm, the average percentage of free vision and the minimum distance to obstacles both decreased from 92.9% to 92.2% and 6 to 20 mm, respectively. This explained that all candidates selected on Pareto front had a minimal *d*7 value allowed by the exploration area (5 mm). The effect of *α*4 and *α*5 angular parameters could not be analyzed most probably because they were correlated (data not shown). In contrast, based on angular parameter *α*6, results could be divided into 4 clusters (Figure 21).

*S*_*v*_ and *S*_*o*_ scores varied monotonically as a function of changes in the linear parameters *L*5, *L*6, and *d*7 (Figure 18, Figure 19, and Figure 20). All 11 candidates selected by Pareto's front had the same set of values for these parameters corresponding to the limits of the exploration area. It should be noted that when *d*7 decreased, the tool body was hidden by the last rotary link when its axis was parallel to the vision axis. The increase of *L*6 decreased the distance to the obstacles (*S*_*o*_) down to the imposed limited of 60 mm during the approach phase. This limit was determined by the fact that the microscope focal length (300 mm), the tool's maximum length (180 mm), and the evaluation path imposed a 60 mm tool travelling in *z*0 axis (40 mm in volume B + 20 mm to reach the start point). The candidates on Pareto's front were also characterized by the sum of *α*4 and *α*5 values ranging between 80 and 100 while this sum ranged from 50 to 120° over the entire exploration area. *S*_*v*_ and *S*_*o*_ scores varied inversely to each other. Their sense of variation depended on *α*6 (Figure 21): by increasing the latter parameter, the distance to obstacles (*S*_*o*_) raised but the vision score (*S*_*v*_) dropped.

### 7.3. Refined Evaluation of Angular Parameters

**Figure 22:**
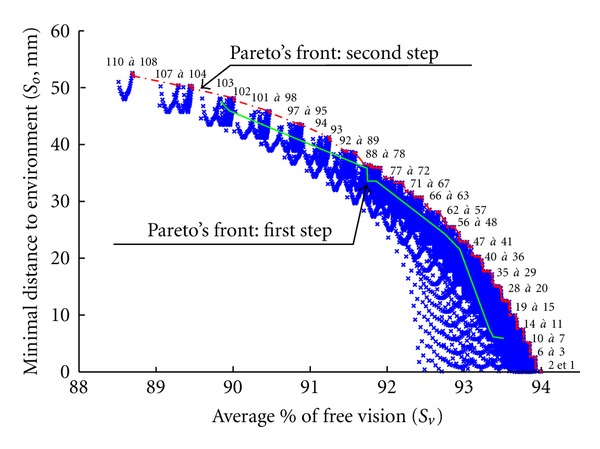
Second step of result optimization: linear parameters *L*5, *L*6, and *d*7 were set at 140, 180, and 5 mm, respectively, and angular parameters *α*4, *α*5, and *α*6 varied in the range of 15 to 60° with a 1° pitch. 9347 candidates meeting all the criteria are plotted. The red-dotted line represents Pareto's front and includes 110 candidates. Pareto's front for the previous step is depicted in green.

The first step of the evaluation did not allow us to fully understand the influence of angular parameters. A second step was designed and undertaken in order to enlarge and to refine the exploration area for *α*4, *α*5, and *α*6. Each of these 3 parameters varied in the range of 15 to 60° with a 1° pitch. The linear parameters *L*5, *L*6, and *d*7 were set at values obtained for the best candidates identified after the first step (i.e., respectively 140, 180, and 5 mm) and fixed by the limits of the exploration area. In this second step of evaluation, 97,336 candidates were assessed and only 9,347 met all the criteria.  Figure 22 shows the distribution of these suitable solutions according to the scores *S*_*v*_ and *S*_*o*_. A Pareto's front (red-dotted line) indicated the best parameter sets. This front included 110 candidates.

Successful candidates were distributed along Pareto's front and formed an arc. Compared to the first step optimization, Pareto's front yielded better results. Indeed, the first front was embedded in the arc. Moreover, the second front was wider than the first one and expanded the suitable solutions to higher scores for *S*_*o*_ and *S*_*v*_. Six top candidates on the front maximizing the score *S*_*o*_ are detailed in Table 3.

Among these solutions, candidate 110 with the highest distance to the external environment was selected to maximize security.

## 8. System Implementation

### 8.1. Components

Linear actuators were selected considering the necessary amplitude of stroke, resolution, and force. A *XYZ* cross table was built with two orthogonal (*X*-*Y*) precision linear stages with a 70 mm travel, orthogonal to a precision linear Z-stage with a 95 mm travel (OWIS: LTM 80P-75-HSM and OWIS: LTM 80P-100-HSM, OWIS GmbH, Staufen, Germany). These linear stages were parallel-mounted, included 2 phase step motors, had hall-effect limit switches, and were economically priced. The resolution of these stages is 0.5 *μm* and the actuating force is 50 N. The choice of rotary actuators was also based on torque, weight, and cost. We chose the same DC micromotor (Faulhaber: 2342S024CR, Faulhaber GmbH, Germany) with magnetic incremental encoder IE2-512 (512 lines per revolution) for all rotary actuators. These were connected to a harmonic drive gearhead with a 50 : 1 reduction ratio (Harmonic Drive: HFUC-8-50-2A-R, Harmonic Drive LLC, Peabody, MA, USA). The two last actuators were placed far from the distal end of the arm in order to keep it as slim as possible. The actuator movements were transmitted to the axis by Bowden cables [39]. This transmission had the advantage of being light and simple to integrate.

### 8.2. Command

**Figure 23:**
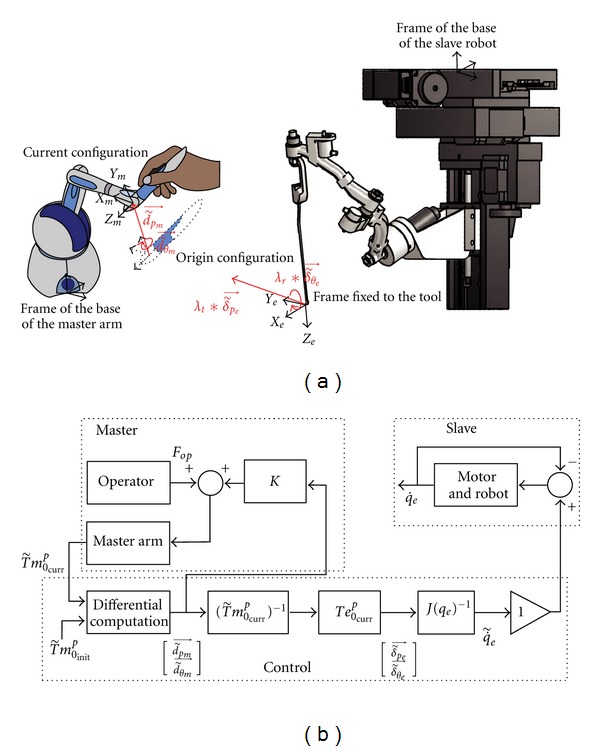
Controller process and structure.

We chose a teleoperated design to command this robot mainly to keep the operating space free around the patient's ear. The Phantom Omni (SensAble Technologies, Inc., Woburn, MA, USA) interface was used as a master arm. In order to have a simple and intuitive master arm, the command was based on a registration between the stylus and the robotic arm frames (Figure 23(a)). This coupling mode resolved the permanent necessity of correspondence between the relative positions of the master and the robotic arms. It could also avoid a potential eye-hand incoordination if an indirect vision feedback was provided (i.e., angled endoscope). A velocity command was implemented. To move the tool, the surgeon defined the stylus position and orientation at the origin by pushing the interface switch before displacing the stylus. A differential computation was performed between the new current stylus position and the original configuration (using (2) described in the Section 6.5). The result was projected and sent to the robotic arm to perform the displacement (Figure 23(b)). A higher amplitude of stylus movement led to a higher speed of the robotic arm. The surgeon released the interface switch to stop the displacement.

## 9. Evaluation

**Figure 24:**
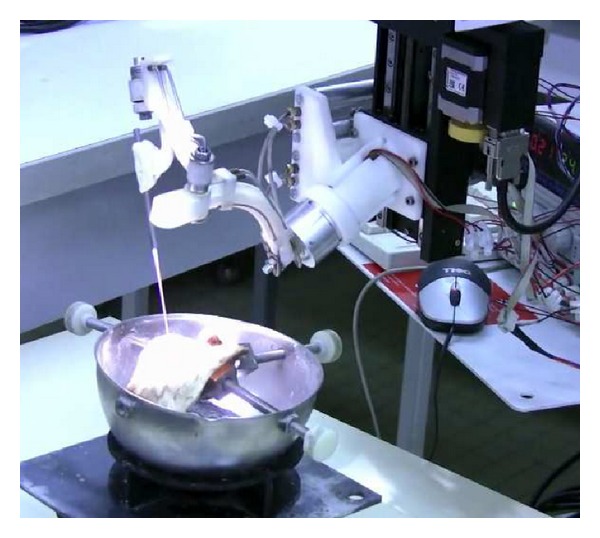
Evaluation of RobOtol in a temporal bone.

The primary objective was to assess the ability of the robot to reach the whole workspace with the tool while preserving the visual field. Achieving this objective would validate the design specifications, kinematic structures, components, and optimization. The secondary objective was to perform a simple task such as stapes removal. The robotic arm was evaluated by a senior ENT surgeon in 3 fresh temporal bones. The robotic device was placed in front of the surgeon. The surgery was performed by a transcanal approach as in clinical practice. To perform the tasks, a 90° angled microhook was mounted on the robotic arm. A direct vision was provided by a microscope with a 400 mm focal lens (Carl Zeiss, Jena, Germany). The master arm was placed under the surgeon's right hand (Figure 24). During the last part of the study we tested the possibility of adding a 30° endoscope in the workspace in order to enhance visualization of the middle ear cavity.

### 9.1. Entire Workspace Accessibility and Visual Field Integrity

#### 9.1.1. Material and Methods

The objective of this step was to reach each of the four quadrants of the tympanic membrane, and after the tympanic membrane displacement, the stapes footplate, and the round window in the middle ear cleft under permanent visual control. Visual field integrity was assessed by peroperative microscope images analysis with ImageJ (http://rsbweb.nih.gov/ij/). Photos of the surgery with the robotic arm were compared with photos extracted from real surgery movies for percentage of free visual field.

#### 9.1.2. Results

**Figure 25:**
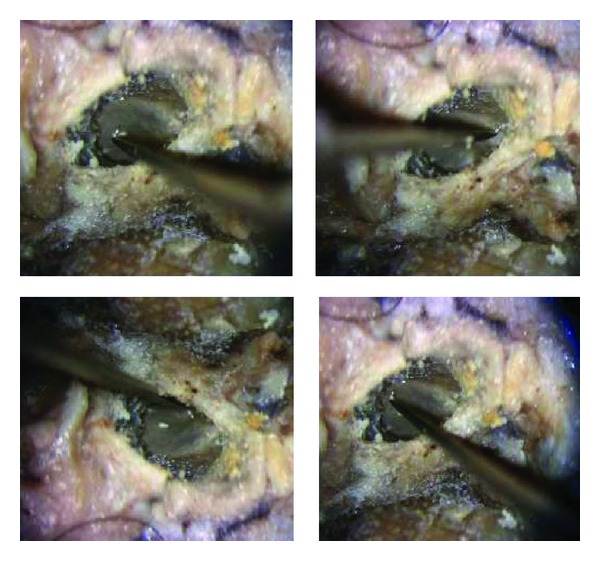
Workspace access. All four quadrants of the tympanic membrane were accessed with a preserved visual field.

 The surgeon was able to teleoperate the tool to reach all the targets with an appropriate accuracy corresponding to the accuracy expected and required during real surgery (Figure 25). In addition, anatomical regions (posterior part of the tympanic cavity: sinus tympani) impossible to reach during real surgery due to the human wrist limitation were accessed with the robotic tool since RobOtol allowed an anterior-posterior direction of the microhook. During the tool displacement singularity configurations were not observed.

**Figure 26:**
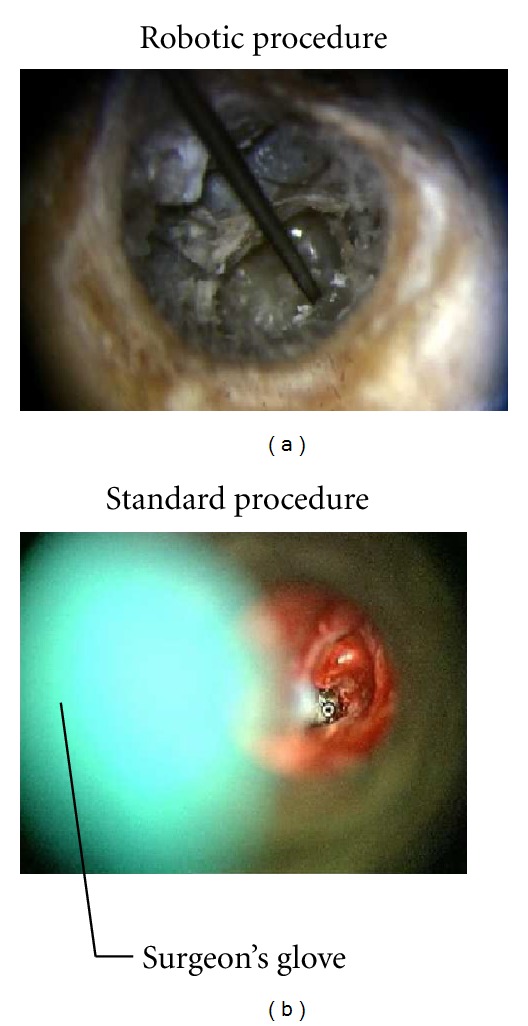
Intraoperative visual field in a robot-assisted technique (a) and in a standard technique (b). Free visual field is enhanced in the robotic procedure.

In unassisted surgical conditions (extracted from real surgery film recordings), the visual field was significantly impaired by the tool and the surgeon's hand holding the tool in the axis of the microscope aligned with the speculum. The measured free visual field represented 63.5% of the speculum diameter. Using the robotic arm, only the tool impaired the visual field. Therefore the measured free visual field was greatly enhanced and represented 91.8% of the speculum diameter (Figure 26).

### 9.2. Stapes Removal and Robot: Endoscopic-Assisted Surgery Evaluation

#### 9.2.1. Material and Methods

**Figure 27:**
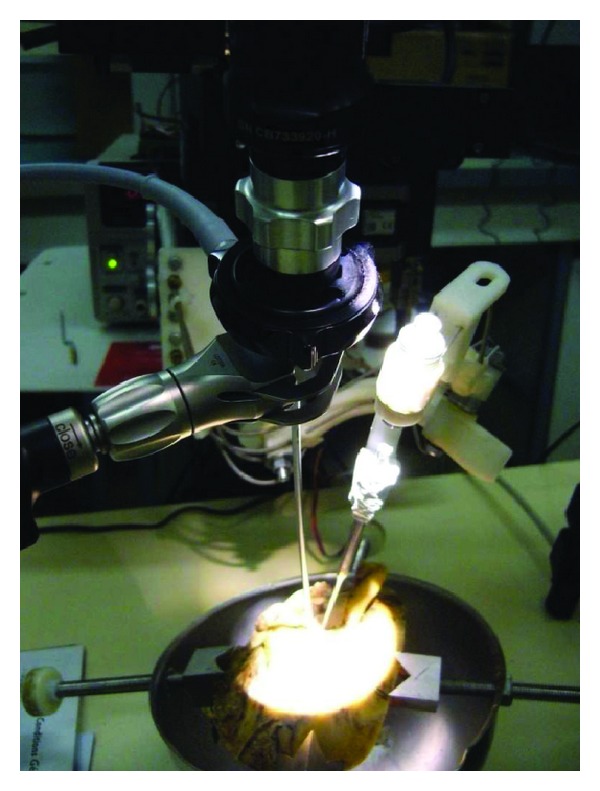
Simultaneous use of the robotic arm and a 4 mm endoscope in the external auditory canal.

Stapes removal was conducted through a transcanal approach with two different means of surgical field visualization. In the first setting, the exposure was obtained with the microscope after scutum lowering. In the second setting, stapes was visualized with a 30° angled, 4 mm diameter endoscope (KARL STORZ GmbH, Tuttlingen, Germany) maintained by a fixed articulated arm (Figure 27). During the endoscopic procedure, no scutum lowering was necessary. Procedure was considered as complete if the stapes could be removed without any damages to surrounding anatomical structures (incus, facial nerve, and cochlea).

#### 9.2.2. Results

**Figure 28:**
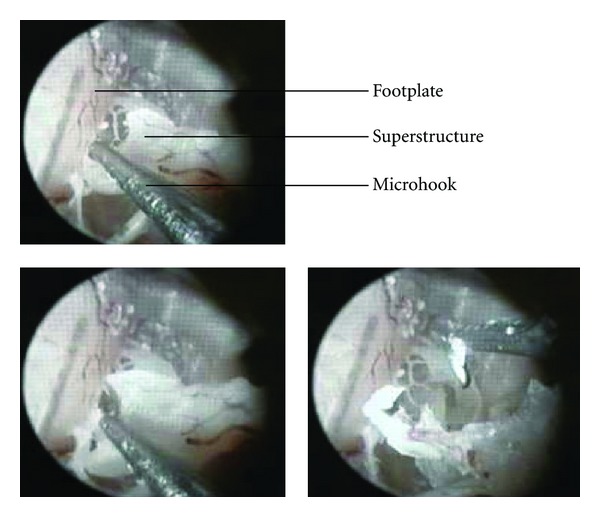
Stapes removal under endoscopic procedure.

Stapedectomy was performed successfully with both the microscope and the endoscope (Figure 28). All other anatomical structures were preserved. The low overall dimensions of the robotic arm allowed easy access and surgical gesture in the middle ear cleft.

### 9.3. Discussion

All evaluation objectives were completed since the surgeon was able to command the robotic arm and reach the entire workspace. The visual field was enhanced in the robot-assisted procedure. A delicate task such as stapes removal could be performed safely. The simultaneous use of the endoscope and the robotic arm was possible. These observations validated the technological design of our prototype. Benefits of robotic assistance for the patients are multiple. This type of assistance will potentially improve the security and lower the risk of lesion to the surrounding anatomical structures. Moreover, surgical functional results could be enhanced by a smoother, a more accurate, and a more reproducible fenestration, prosthesis positioning and crimping. Finally, the robotic-assisted procedure will be potentially faster and less invasive since the procedure can be simplified. The other issue in middle ear surgery is the operating field exposure. Vision provided by the microscope is aligned with the external ear canal which has a small diameter (10 to 6 mm of diameter). Wider exposure is possible but requires ear canal bone drilling and could hamper tympanic membrane closing. To enhance dexterity and reduce tremor, surgeons have to hold the instruments closer to the tip and to stabilize their hands on patient's head around the speculum. Thus, up to one-third of this small visual field can be hidden by the surgeon's hand and tools. In contrast, the robotic arm holds the tool far from the tip and interferes much less with the vision. Another possibility tested was the use of an endoscope with the robotic arm. Endoscopes are used in middle ear surgery to explore the middle ear cleft and can expose all the recesses thanks to the angled optical lens. They are usually used for diagnostic or control during the procedure rather than for surgical treatment because the angled vision hampers the eye-hand coordination. Our prototype and command settings offer the possibility of a referential readjustment and overcome the eye-hand shift issue. With our command settings, it will make no difference for the surgeon to work with a straight, 30° or 70° angled endoscope. Moreover, the arm dimensions are small enough to travel all around the endoscope to perform the task. Thus the standard otosclerosis procedure requiring a scutum lowering can be refined to a less invasive technique by keeping the complete bone frame around the tympanic membrane.

## 10. Conclusion and Future Works

The specifications of the otologic robot were defined to take into account all constraints of middle ear surgery: Basic data such as the reachable workspace size and the tool-organ interaction forces collected experimentally and a qualitative reflection on the particular context of otologic surgery and its ergonomics. Based on these specifications, a robot kinematics comprising a cross table at the base and a wrist with remote center of motion was selected. The geometric dimensions of the structure were parameterized and optimized using a specific method. The evaluation of robot candidates required to model the task in all its aspects: collision avoidance with extracorporeal environment, reachable workspace, and visual field preservation. The optimization study revealed the influence of the linear geometrical parameters on the visual field preservation and on the robot integration with the extracorporeal environment. Finally, we evaluated the first prototype in human temporal bone showing the validity of our calculations and choices. Stapes removal was performed in human temporal bones under microscopic vision. The surgical procedure could be improved with the use of an endoscope. The next step is to design a full set of tools for the robotic arm in order to perform the full procedure. Duration and tool-organ interaction force will then be compared between manual and robotic-assisted technique per surgeons with different levels of experience. Our design process can be applied to other robotic systems in the surgical field.

## Figures and Tables

**Table 1:** Exploration area of the parameters.

	*α*4	*L5 *	*α*5	*α*6	*d7 *	*L6 *
Min. values	25°	90 mm	25°	15°	5 mm	130 mm
Max. values	60°	140 mm	60°	60°	30 mm	180 mm
Pitch	5°	10 mm	5°	5°	5 mm	10 mm

**Table 2:** Parameter values for 11 candidates selected by the Pareto's front.

Sol. no.	*α*4 (°)	*L*5 (mm)	*α*5 (°)	*α*6 (°)	*d*7 (mm)	*L*6 (mm)	Vision score *S*_*v*_ (%)	Distance score *S*_*o*_ (mm)
1	30	140	60	50	5	180	93,511	5,995
2	50	140	50	50	5	180	93,376	6,247
3	40	140	40	50	5	180	93,327	7,526
4	40	140	55	45	5	180	92,955	21,29
5	50	140	45	45	5	180	92,941	21,747
6	50	140	40	45	5	180	92,752	24,442
7	35	140	55	40	5	180	91,861	33,785
8	50	140	40	40	5	180	91,748	33,788
9	55	140	40	40	5	180	91,741	36,087
10	35	140	55	35	5	180	89,964	46,21
11	55	140	35	35	5	180	89,841	47,913

**Table 3:** Values of the 6 top candidates after the second step of result optimization.

Solution no.	*α*4	*α*5	*α*6	Visual score	Distance score
110	32°	58°	33°	88.691%	52.6 mm
109	31°	59°	33°	88.696%	52.4 mm
108	30°	60°	33°	88.701%	52.04 mm
107	30°	59°	34°	89.29%	50.42 mm
106	31°	59°	34°	89.44%	50.33 mm
105	32°	59°	34°	89.464%	50.23 mm
104	31°	60°	34°	89.467%	49.85 mm
